# A Measurement Invariance Analysis of the Interpersonal Needs Questionnaire and Acquired Capability for Suicide Scale in Autistic and Non-Autistic Adults

**DOI:** 10.1089/aut.2019.0055

**Published:** 2020-09-03

**Authors:** Mirabel K. Pelton, Hayley Crawford, Ashley E. Robertson, Jacqui Rodgers, Simon Baron-Cohen, Sarah Cassidy

**Affiliations:** ^1^ Centre for Intelligent Healthcare, Coventry University, Coventry, United Kingdom.; ^2^ School of Psychology, University of Birmingham, Birmingham, United Kingdom.; ^3^ CMHWR and Mental Health and Wellbeing Unit, Division of Health Sciences, Warwick Medical School, University of Warwick, Warwick, United Kingdom.; ^4^ Faculty of Health and Life Sciences, Coventry University, Coventry, United Kingdom.; ^5^ Population Health Sciences Institute, Sir James Spence Institute, Newcastle University, Royal Victoria Infirmary, Newcastle, United Kingdom.; ^6^ Autism Research Centre, University of Cambridge, Cambridge, United Kingdom.; ^7^ Cambridge Lifetime Asperger Syndrome Service (CLASS), Cambridgeshire and Peterborough NHS Foundation Trust, Cambridge, United Kingdom.; ^8^ School of Psychology, University of Nottingham, Nottingham, United Kingdom.

**Keywords:** suicide, measurement, interpersonal theory, burdensomeness, belonging, suicidal capability

## Abstract

***Background:*** Autistic adults are more likely to engage in suicidal thoughts and behaviors, but there is little research to explore the underlying reasons. It is unclear whether self-report suicide scales that have been designed for non-autistic people accurately measure suicide risk constructs in autistic people. Therefore, this study explored, for the first time, whether the measurement properties of the self-report scales of the Interpersonal Theory of Suicide are equivalent in autistic and non-autistic adults.

***Methods:*** In this study, responses from 342 autistic and 353 non-autistic people on the Interpersonal Needs Questionnaire-10 (INQ-10) and Acquired Capability for Suicide Scale–Fearlessness about Death (ACSS–FAD) were compared by using measurement invariance analysis. Data were gathered through an online cross-sectional survey of the self-report measures.

***Results:*** Results suggest that measurement properties of the INQ-10 were different in autistic people. Autistic characteristics, such as different theory of mind and preference for concrete language, may have led the scale items to load differently on the factors in the autistic group than in the non-autistic group. The measurement properties of the ACSS–FAD were invariant between autistic and non-autistic people.

***Conclusions:*** Scores on the INQ-10 cannot be meaningfully compared between autistic and non-autistic people due to different measurement properties. Future research could explore how autistic people experience the concepts of burdensomeness and belonging, to consider how measures could accurately capture this. This would allow researchers to explore the role of these constructs in the development of suicidal thoughts and behaviors in autistic people. Clinicians should be aware that suicide risk factors may present differently in autistic people. Scores on the ACSS-FAD can be meaningfully compared, but the negatively worded scale items may pose similar response difficulties to autistic and non-autistic people.

## Lay summary

**What was the purpose of this study?:**

The purpose of this study was to explore whether two questionnaires designed for non-autistic people create response difficulties for autistic people. If autistic people experience difficulties interpreting the questions, this can mean that the scales do not work in the same way, which means that scores between autistic and non-autistic people cannot be compared. This is important, as these questionnaires are designed to measure risk factors for suicide: feeling like a burden on others, feeling as if you don't belong, and not fearing death. We know that for non-autistic people these feelings increase the risk of suicide, but we do not yet know whether this is the case for autistic people.

**What did the researchers do?:**

We collected questionnaire responses online from 342 autistic people and 335 non-autistic people. We then used statistical analyses to identify whether the questionnaires operated differently in autistic and non-autistic people. A steering group of autistic adults identified which questions were likely to be interpreted differently by autistic people.

**What were the results of the study?:**

We found that on the questionnaire designed to test feeling like a burden, autistic people appeared to have interpreted all items differently. This means that we cannot compare scores on this scale between autistic and non-autistic people. We found that on the questionnaire designed to test feelings of not belonging total scores could not be compared, because autistic people seemed to have interpreted two items differently. On the questionnaire measuring a reduced fear of death, total scores can be compared as all items appear to have been interpreted similarly between autistic and non-autistic people.

**What do these findings add to what was already known?:**

These findings provide evidence that the questions in these questionnaires may be differently interpreted by autistic people, meaning that scores cannot be meaningfully compared. This could mean that feelings of burdensomeness and belonging are experienced differently by autistic people.

**What are the potential weaknesses in the study?:**

This study has not investigated whether other factors, such as other neurodevelopmental conditions, may influence how someone responds to these scales. Also, future research should also include the experiences of autistic people with intellectual disability.

**How will these findings help autistic adults now or in the future?:**

These findings will alert clinicians immediately to the fact that autistic people may show risk factors for suicide differently from non-autistic people. They may need to ask different questions or look out for different signs. In future, this may help us to understand how different factors may contribute to suicidal thoughts and behaviors for autistic and non-autistic people.

## Introduction

Research consistently reports more frequent suicidal thoughts and behaviors^1^ and significantly higher rates of death by suicide among autistic^*^ compared with non-autistic people.^2,3^ However, there is a lack of research to explore how proximal risk factors may lead to the development of suicidality in autistic people, and there are no validated tools to identify such constructs and assess risk severity.^4^ This study explores the appropriateness and measurement properties of the self-report scales of one of the most highly cited models of suicide—the Interpersonal Theory of Suicide (ITS).^5,6^ This affords the opportunity to compare the extent to which the ITS operates similarly in autistic and non-autistic people. Understanding differences will inform suicide interventions that meet the needs of autistic people and identify autism-specific risk markers.

The ITS proposes that, in any population group, the unmet need for social connections (termed “thwarted belonging”) and unmet need for self-worth (termed “perceived burdensomeness”) together lead to suicidal thoughts.^5,6^ Individuals who have repeatedly experienced painful and frightening events may develop *suicidal capability*—a habituation to fear and pain that enables suicidal thoughts to be actioned.^5,6^ The concepts of burdensomeness and belonging are measured by distinct subscales of the *Interpersonal Needs Questionnaire* (INQ)^7^, and suicidal capability is measured by the 7-item *Acquired Capability for Suicide Scale***–***Fearlessness about Death* (ACSS**–**FAD).^8^ The INQ and ACSS**–**FAD have been validated in U.S. undergraduates, psychiatric outpatients, and adolescent inpatients^9^ but their measurement properties have not yet been explored in an autistic sample. This is the aim of the current study.

The ITS may be of particular relevance to autistic adults, as it highlights the importance of constructs such as autonomy for quality of life ^10,11^ and the risks to mental health of burdensomeness^12,13^ and social exclusion,^14,15^ which have been identified as important issues for autistic people. In both childhood and adulthood, autistic people experience significantly more traumatic life events than non-autistic people, such as being bullied or exploited.^16,17^ Our earlier research reported that, in a non-clinical sample of young adults, the ITS hypotheses were upheld even at high levels of autistic traits.^18^ Our recent survey showed that autistic people reported more suicidal thoughts and behaviors and stronger feelings of burdensomeness and thwarted belonging than non-autistic adults. However, in the autistic group, the association between each of thwarted belonging and perceived burden with suicidal thoughts and behaviors was significantly attenuated compared with the non-autistic group.^19^

One possible explanation for this attenuation is that autistic characteristics influenced responses to the scale items and, thus, the scale measurement properties in the autistic group. Autistic adults could have less confidence than non-autistic people to infer how others feel about them (termed “theory of mind”),^20^ infer their internal emotional state (termed “alexithymia,” which is more common in autistic than non-autistic people),^21^ and may prefer concrete terms to describe social emotional states.^22^ Thus, autistic people may endorse a lower score on the burdensomeness sub-scale than non-autistic people, because they are unsure how others feel about them, rather than because they feel less of a burden. This could result in a different factor structure, reduced convergent validity, and reduced strength of correlations between the variables of interest, which, in turn, could explain the attenuated associations in the autistic group. Measurement invariance analysis can quantitatively identify differences in measurement structure between groups with increasing stringency, which allows researchers to explore the extent to which results between groups are comparable (invariant) or different (non-invariant).^23^ Summarized in Table 1, we hypothesized, with our autistic steering group (described in Methods section), that, compared with non-autistic people, autistic people would (i) interpret all items of the INQ-10 differently given differences in theory of mind and preference for concrete language; and (ii) interpret four of the ACSS-FAD scale items differently due to difficulties with negatively worded questions and preference for concrete language.

**Table 1.** Detailed Hypotheses of Item Invariance for Interpersonal Needs Questionnaire-10 and Acquired Capability for Suicide Scale**–**Fearlessness about Death

Item number	INQ items	How would autistic/non-autistic adults answer?	Consensus within design group?	Reason
	Construct: Perceived burdensomeness			
1	These days, the people in my life would be better off if I were gone	Differently (non-invariant)	No consensus	These items rely on the non-autistic theory of mind
2	These days, the people in my life would be happier without me		Consensus	
3	These days, I think my death would be a relief to the people in my life		No consensus	
4	These days, I think the people in my life wish they could be rid of me		No consensus	
5	These days, I think I make things worse for people in my life		No consensus	
	Construct: Thwarted belonging			
6	These days, I feel like I belong	Differently (non-invariant)	Consensus	Non-concrete language: “I belong”
7	These days, I am fortunate to have many caring and supportive friends		Consensus	Non-concrete language: “many,” “supportive”
8	These days I feel disconnected from other people		No consensus	Non-concrete language: “disconnected”
9	These days, I often feel like an outsider at social gatherings		Consensus	Overlap with autistic characteristics
10	These days I am close to other people		Consensus	Non-concrete language “close” and “other” people
	ACSS**–**FAD items			
	Construct: Reduced fear of death			
1	The fact that I am going to die does not frighten me at all	Differently (non-invariant)	Consensus	Negatively worded item leading to difficulty identifying correct (negative) response on scale
2	The pain involved in dying frightens me	Same (invariant)	Consensus	Clear item wording
3	I am very much afraid to die	Differently (non-invariant)	No consensus	Non-concrete language “very much”
4	It does not make me nervous when I talk about death	Differently (non-invariant)	Consensus	Negatively worded item leading to difficulty identifying correct (negative) response on scale. This item was identified as most difficult to identify correct response.
5	The prospect of my own death arouses anxiety in me	Same (invariant)	Consensus	Clear item wording
6	I am not disturbed by death being the end of life, as I know it	Differently (non-invariant)	Consensus	Negatively worded item leading to difficulty identifying correct (negative) response on scale
7	I am not at all afraid to die	Differently (non-invariant)	Consensus	Non-concrete language “at all”

ACSS**–**FAD, Acquired Capability for Suicide Scale**–**Fearlessness about Death; INQ, Interpersonal Needs Questionnaire.

## Methods

### Involvement of autistic adults

Our steering group of autistic adults (one male, one female) identified the focus of this study in our first meeting when they reported difficulty interpreting scale items. This group comprises autistic adults without intellectual disability recruited by open invitation to local autism groups. The group meets two to three times a year to provide feedback on each stage of the research process. In this study, they reviewed materials, suggested modifications to survey wording including clear risk signposting, and guided detailed hypotheses and analysis strategy.

### Participants

Data were retained from 343 autistic and 335 non-autistic participants from a larger survey dataset of online cross-sectional and repeated measures undertaken in Qualtrics.^19^ Participants provided informed consent, were warned about the content of questions in each section, were advised that they may skip sections if they wished, were prompted to take breaks, and were given information about support services. This study received ethical approval from Coventry University ethics committee.

Participants self-reported autism diagnosis by a specified medical professional, and mean Autism Quotient scores were within clinical levels. Autistic participants were recruited through the Cambridge Autism Research Database, Autistica Discover network, social media, and local and national autism organizations. Non-autistic participants were recruited through the University of Cambridge Psychology Database, suicide-focused websites, Coventry University research participation scheme, and opportunity sampling to match group size, mean age, and gender frequency with the autistic group. The samples self-selected whether to respond to either or both of the INQ-10 and ACSS**–**FAD (Table 2).

**Table 2.** Descriptive Statistics and Sample Characteristics for Autistic and Non-Autistic Samples

	INQ sample autistic* n* = 343, 58.6% female	INQ sample not autistic* n* = 335, 64.5% female	ACSS***–***FAD sample autistic* n* = 343, 58.5% female	ACSS***–***FAD not autistic* n* = 332, 64.8% female	Significant differences between autistic and non-autistic group^*^
Age (mean/SD)	42.1 (13.6), 18–90	41.5 (15.7) 18–73	42.2	41.6 (15.7), 18–90	*U* = 54,212.5, *p* = 0.31
AQ-S (mean/SD)	91.1 (11.45)	60.9 (12.7)	91.08 (11.45)	60.9 (12.7)	*U* = 4851, *p* < 0.001
Perceived burden subscale (mean/SD)	14.87 (8.02)	8.70 (6.04)	14.87 (8.03)	8.71 (6.06)	*U* = 27,602.5, *p* < 0.001
Thwarted belonging (mean/SD)	25.94 (6.77)	16.54 (8.01)	25.96 (6.78)	16.59 (8.04)	*U* = 21,801.5, *p* < 0.001
Capability for suicide	16.48 (7.18)	16.17 (6.94)	16.50 (7.18)	16.17 (6.94)	*U* = 54,533.5, *p* = 0.34
Reporting at least one past suicide attempt, *n* (%)	131 (38.3)	35 (10.4)	131 (38.2)	35 (10.5)	χ^2^ (1) = 58.36, *p* < 0.001
Full-time employed, *n* (%)	97 (28.3)	141 (42.1)	98 (28.6)	140 (42.2)	χ^2^ (1) = 13.66, *p* < 0.001
Living with support, *n* (%)	85 (24.8)	41 (12.2)	85 (24.8)	41 (12.3)	χ^2^ (1) = 17.17, *p* < 0.001
Currently diagnosed with at least one mental illness, *n* (%)	240 (70)	98 (29.3)	239 (69.7)	97 (29.2)	χ^2^ (1) = 110.48, *p* < 0.001
Diagnosed with at least one neurodevelopmental condition (in addition to autism), *n* (%)	105 (30.6)	28 (8.4)	105 (30.6)	27 (8.1)	χ^2^ (1) = 54.19, *p* < 0.001
% with post-graduate degree, *n* (%)	121 (35.3)	151 (45.1)	122 (35.6)	151 (45.5)	χ^2^ (1) = 7.08, *p* = 0.008

^*^ Differences between groups are based on the ACSS**–**FAD sample.

#### Measures of interest

The INQ-10^24^ is a 10-item scale measuring *thwarted belonging* and *perceived burden*. Our steering group revised instructions to clarify the meaning of the scale instructions for autistic people (agreed with the scale author before administration): “Please read the items below.” Click on the option that best describes how you have been feeling. Where the questionnaire refers to “these days” please consider how you have been feeling in general over the past 2 weeks. Items include “these days I feel like I belong” and “these days I think the people in my life would be happier without me” with a 7-point response scale from “strongly agree” to “strongly disagree,” with higher scores indicating stronger feelings of thwarted belonging and perceived burden. The INQ is reported to measure the same latent traits in U.S. undergraduates, psychiatric outpatients,^9^ older adults,^7,25^ and men and women.^26^ The 10-item version employed here demonstrates a more consistent model fit and predictive validity than other versions^9^ (Non-autistic burdensomeness subscale α = 0.93, autistic = 0.92, non-autistic belonging subscale α = 0.90, autistic = 0.86).

ACSS**–**FAD^8^ is a 7-item scale measuring *suicidal capability* with a response scale from 0 “not at all like me” to 4 “very like me,” with higher scores indicating higher suicidal capability. Items include “the prospect of my own death arouses anxiety in me” and “I am not at all afraid to die,” with items 2, 3, and 5 describing fear of death and, thus, reverse coded. The ACSS**–**FAD has been validated in undergraduate samples, measures the same latent traits in men and women, and demonstrates convergent/divergent validity with associated constructs in psychiatric samples^8^ (Non-autistic α = 0.85, autistic = 0.84).

#### Demographic variables

Autism Quotient Short Form (AQ-S)^27^ measured *autistic characteristics*. The AQ-S is a 28-item subset of the AQ-50 and includes items such as “it does not upset me if my daily routine is disturbed” and “I find it easy to work out what someone is thinking or feeling,” with a 4-item response scale from 1 “definitely agree” to 4 “definitely disagree.”^27^ The AQ-S demonstrates the same latent traits in clinical and non-clinical groups^28^ (α = 0.88 non-autistic, 0.87 autistic).

Suicide Behaviors Questionnaire—Revised, item 1 measured *Lifetime suicidal thoughts and behaviors* asking “Have you ever thought about or attempted to kill yourself?” Participants self-reported previous suicidal behavior by choosing one of six possible responses from “never” to “I have attempted to kill myself and really hoped to die.”^29^ Item 1 demonstrates comparable measurement properties in autistic and non-autistic adults.^4,30^

### Analysis strategy

#### Establishment of a baseline model

Analyses were undertaken in AMOS 25. Confirmatory factor analysis (CFA) tested how well previously published models account for the correlations between variables (termed “model fit”). Good model fit was assessed by using fit indices: comparative fit index (CFI) of at least 0.95 (excellent or above 0.9 acceptable), root mean square of approximation of <0.05 (excellent or <0.1 acceptable), standardized root mean square residual of <0.09, p of Close Fit of at least 0.05, and chi-square/degrees of freedom of <3 (excellent or <5 acceptable).^31^ Convergent validity was assessed by using a score of more than 0.5 of average variance extracted, which measures variance captured by items and variance due to error.^32^

In case of poor model fit, alternative models were tested by using: (i) alternative published models or (ii) suggested modification indices, our hypotheses, and review of item meaning. Models were then tested in the autistic group. The best fitting “baseline” model in each group was taken forward for measurement invariance analysis.

### Measurement invariance analysis

Multi-group CFA allows researchers to test the extent to which measurement properties are equivalent (invariant) across groups.^23^ Termed “measurement invariance analysis,” increasing parameters, such as factor loadings or error terms, are held equal in both groups and the model is tested for significant change (degradation) in fit indices. A significant degradation in model fit indices indicates lack of evidence for measurement invariance between the groups, suggesting that the measure operates significantly differently in each group (non-invariant).

First, the configural model tests whether the sets of items measure the same latent construct in both groups with no equality constraints. In the case of configural invariance, factor loadings are subsequently constrained equal between groups to test whether scale items associate similarly with each factor in each group (“metric invariance”). In the case of metric non-invariance (factor loadings significantly different between groups), the individual non-invariant items are identified by constraining the factor loadings for each item in turn. In the case of metric invariance, factor loadings and intercepts are subsequently constrained equal to test whether total scores consist of similar individual item scores in each group (“scalar invariance”). In the case of scalar invariance, error terms and error co-variances are constrained equal to test whether the scale items measure the same latent construct with comparable measurement error (“residual” or “strict invariance”).^33^ To consider a tool measurement invariant between two groups, scalar invariance has to be demonstrated, as this suggests that mean scores will be broadly comparable between groups.^33^

A reduction in CFI of <0.01 alongside non-significant change in chi-square indicate measurement invariance, suggesting that the items of the tool operate similarly between the two groups.^34^ Greater differences in fit statistics indicate lack of evidence for measurement invariance, suggesting that the items operate significantly differently between the groups (non-invariant). For example, lack of evidence for metric invariance indicates that the groups attribute different meaning to the items, and they are therefore metric non-invariant.

## Results

### Interpersonal Needs Questionnaire-10

#### Baseline model

**FIG. 1.**
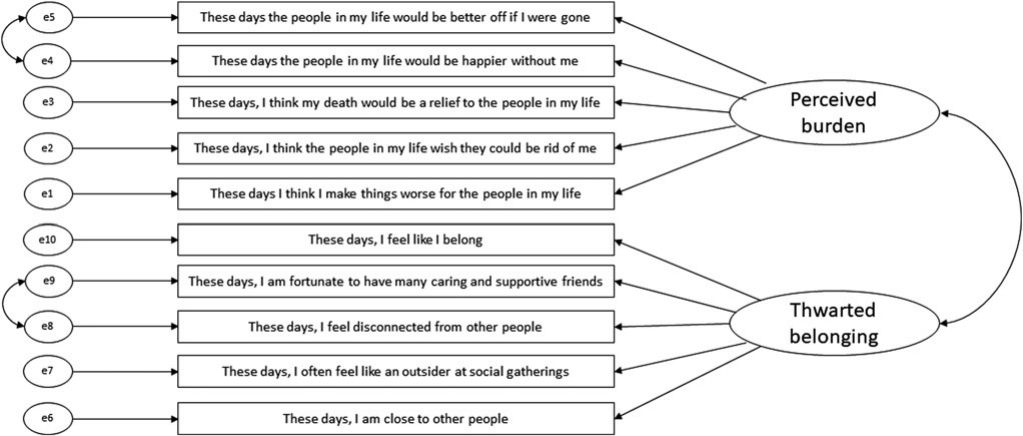
Interpersonal Needs Questionnaire-10 model 2 retained for measurement invariance analysis in autistic and non-autistic groups.

Data were screened for outliers and normality. In the non-autistic group, data were significantly multivariate kurtotic (standardized kurtosis = 59.99, >5)^23^ and four burdensomeness items had significant univariate kurtosis (>7).^35^ Kurtosis represents significant problems in tests of variance and co-variance structures, resulting in inflated chi-square statistic.^23,36^ Thus, given sufficient sample size (at least 10 × number of parameters estimated)^37^ asymptotic distribution-free (ADF) estimation was used. In the autistic group, data were normally distributed so maximum likelihood (ML) estimation was used. We tested a two-factor solution (five items each) with no co-varied error terms (model 1) and with two error terms co-varied (model 2) as in previous studies^7,9^ and suggested by similar item meaning. As shown in Table 3, model 2 (in Fig. 1) achieved at least acceptable fit across four indicators in the non-autistic and autistic groups and was retained for invariance analysis.

**Table 3.** Model Fit Indices from Interpersonal Needs Questionnaire-10 Baseline Confirmatory Factor Analysis

Non-autistic group	χ^2^/df	CFI	SRMR	RMSEA	pClose
Model 1	**2.903**	0.843	0.118	**0.075**	0.008
Model 2	**2.239**	**0.904**	0.119	**0.061**	**0.160**
Autistic group					
Model 1	**2.939**	0.**969**	**0.050**	**0.075**	0.021
Model 2	**2.595**	**0.976**	**0.045**	**0.068**	0.046

Model 1 = two factor model, no error terms co-varied. Model 2 = previously published model with two pairs of error terms co-varied.^7,9^ Bold indicates adequate or excellent model fit: CMIN/df <5, CFI >0.9, SRMR <0.09, RMSEA <0.1, pClose >0.05. Model run by using asymptotic distribution-free estimation in the non-autistic group and maximum likelihood estimation in the autistic group.

CFI, comparative fit index; CMIN/df, chi-square/degrees of freedom; pClose, p of Close Fit; RMSEA, root mean square of approximation; SRMR, standardized root mean square residual.

### Measurement invariance

Table 4 shows results of measurement invariance analysis. The configural model (1) was estimated in both groups by using ADF estimation to reflect the theorized non-normal distribution of INQ experiences. This model suggested degradation of fit with respect to baseline models despite no factor cross-loadings indicated. Given the difference in distribution in burdensomeness in each group, we explored factor differences. The measurement model (2a) demonstrated significant degradation in model fit as did the constraint of each factor in turn (perceived burden 2b) and (thwarted belonging 2c). The constraint of each item of the burdensomeness factor resulted in significant degradation of model fit, indicating metric non-invariance across all items of the burdensomeness scale (models 2d–2h). Constraint of each item of the belonging factor indicated that two items (models 2i and 2j) resulted in significant model degradation, thus suggesting metric non-invariance for these two items of the belonging scale. However, three items (models 2k–2m) did not result in significant degradation of model fit, suggesting evidence for metric invariance for these three items of the belonging scale. Overall, we did not find evidence of metric invariance so stricter tests were not undertaken.

**Table 4.** Summary of Goodness-of-Fit Statistics for Multigroup Invariance Tests for the Interpersonal Needs Questionnaire-10

Model no	Description	Contrast	χ^2^	df	χ^2^ Δ	df Δ	Δ p	CMIN/df	CFI	Δ CFI	SRMR	RMSEA	pClose
**1**	**Configural model (unconstrained)**		**159.45**	**64**				**2.491**	**0.883**		**0.119**	**0.047**	**0.695**
**2a**	**Measurement model (all factor loadings constrained equal)**	**2a vs. 1**	**262.733**	**74**	**77.01**	**10**	**<0.001**	**3.550**	**0.769**	**0.082**	**0.091**	**0.695**	**0.010**
**2b**	**Burden factor (burden subscale factor loadings constrained equal)**	**2b vs. 1**	**219.314**	**69**	**43.419**	**5**	**<0.001**	**3.178**	**0.816**	**0.055**	**0.097**	**0.057**	**0.182**
**2c**	**Belong factor (belong subscale factor loadings constrained equal)**	**2c vs. 1**	**185.902**	**69**	**26.452**	**5**	**<0.001**	**2.694**	**0.857**	**0.034**	**0.169**	**0.050**	**0.480**
**2d**	**Configural model + “these days I make things worse for the people in my life”**	**2d vs. 1**	**197.926**	**65**	**38.476**	**1**	**<0.001**	**3.045**	**0.838**	**0.045**	**0.087**	**0.055**	**0.166**
**2e**	**Configural model + “these days I think the people in my life wish they could be rid of me”**	**2e vs. 1**	**191.685**	**65**	**32.235**	**1**	**<0.001**	**2.949**	**0.845**	**0.038**	**0.077**	**0.054**	**0.234**
**2f**	**Configural model + “these days I think my death would be a relief to the people in my life”**	**2f vs. 1**	**213.713**	**65**	**54.263**	**1**	**<0.001**	**3.288**	**0.818**	**0.065**	**0.097**	**0.058**	**0.058**
**2g**	**Configural model + “these days, I think the people in my life would be happier without me”**	**2g vs. 1**	**204.115**	**65**	**33.963**	**1**	**<0.001**	**3.140**	**0.830**	**0.053**	**0.087**	**0.056**	**0.113**
**2h**	**Configural model + “these days, the people in my life would be better off if I were gone”**	**2h vs. 1**	**208.415**	**65**	**38.864**	**1**	**<0.001**	**3.206**	**0.825**	**0.058**	**0.091**	**0.057**	**0.085**
**2i**	**Configural model + “these days, I am close to other people”**	**2i vs. 1**	**169.249**	**65**	**37.822**	**1**	**<0.001**	**2.604**	**0.873**	**0.010**	**0.126**	**0.049**	**0.578**
**2j**	**Configural model + “these days, I feel like an outsider at social gatherings”**	**2j vs. 1**	**175.772**	**65**	**16.321**	**1**	**<0.001**	**2.704**	**0.865**	**0.018**	**0.153**	**0.050**	**0.469**
2k	Configural model + “these days, I feel disconnected from people”	2k vs. 1	161.008	65	1.558	1	0.212	2.477	0.883	0	0.122	0.047	0.710
2l	2k + “these days I am fortunate to have many caring and supportive friends”	2l vs. 2k	165.262	66	4.254	1	0.039	2.504	0.879	0.004	0.129	0.047	0.685
2m	2l + “these days I feel like I belong”	2m vs. 2l	169.229	67	3.967	1	0.046	2.526	0.875	0.004	0.138	0.048	0.664

Bold indicates non-invariant measurement model and individual items (factor loadings significantly different between groups indicating metric non-invariance).

### Acquired capability for suicide scale: fearlessness about death

#### Baseline models

**FIG. 2.**
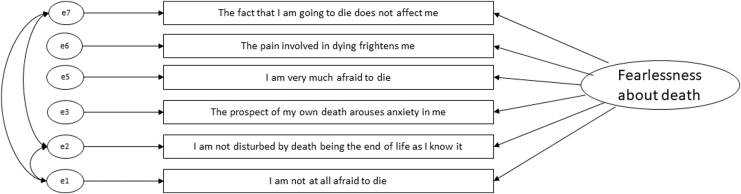
Acquired Capability for Suicide Scale**–**Fearlessness about Death model 4 retained for measurement invariance analysis in autistic and non-autistic groups.

Data were normally distributed so ML estimation was used. Model 1 tested a single factor structure (seven items) consistent with previous literature; model 2 added two co-varied error terms; and model 3 included a subset of those educated to at least degree level.^8^ Due to poor convergent validity, we removed item 4 (the weakest loading item) with a factor loading of 0.41. This item—“It does not make me nervous when people talk about death”—was viewed as the most confusing by our steering group. We co-varied error terms of other negatively worded items in line with hypotheses/modification indices (model 4). Table 5 shows that model 4 achieved good fit in all fit indices in the non-autistic and autistic groups and was retained for measurement invariance analysis (Fig. 2).

**Table 5.** Baseline Models of the Acquired Capability for Suicide Scale**–**Fearlessness about Death in Autistic and Non-Autistic Adults

Not autistic group	CMIN/df	CFI	SRMR	RMSEA	pClose	AVE
Model 1	11.813	0.866	**0.079**	0.181	0.000	0.486
Model 2	8.836	**0.917**	**0.076**	0.154	0.000	0.467
Model 3	8.588	0.865	**0.080**	0.177	0.000	0.475
Model 4	**1.541**	**0.997**	**0.021**	**0.040**	**0.563**	**0.509**
Autistic group						
Model 1	7.177	**0.929**	**0.063**	0.135	0.000	0.489
Model 2	7.195	**0.939**	**0.060**	0.135	0.000	0.482
Model 3	3.773	**0.955**	**0.050**	0.109	0.001	**0.513**
Model 4	**2.018**	**0.995**	**0.020**	**0.055**	**0.372**	**0.545**

Model 1 = all scale items, no co-variances.^8^ Model 2 = model 1 with error terms co-varied “The fact that I'm going to die does not affect me” and “I am not at all afraid to die” and “The pain involved in dying frightens me” and “I am very much afraid to die”. Model 3 = all scale items those endorsing at least undergraduate level education (*n* = 242 not autistic, *n* = 233 autistic). Model 4 = exploratory model with item 4 removed “It does not make me nervous when people talk about death” and error terms co-varied between other negatively worded items. Bold indicates adequate or good model fit. CMIN/df <5, CFI >0.9, SRMR <0.09, RMSEA <0.1, pClose >0.05. AVE (Average Variance Extracted) >0.5 for adequate convergent validity.

#### Measurement invariance

Table 6 shows the results of measurement invariance analysis. The configural model (1) showed similar fit to the baseline model and no degradation in model fit. The measurement model (2) with constrained factor loadings also showed similar fit and no degradation in model fit, as did the scalar model with intercepts also constrained (3). Finally, with error terms and co-variances also constrained, the residual model (4) indicated reduced and marginally significant degradation of model fit. Constraining error terms and co-variance for the individual negatively worded items showed non-significant degradation in model fit. This analysis suggests that the ACSS**–**FAD meets criteria for scalar invariance between the groups, with evidence for residual or strict invariance for negatively worded items.

**Table 6.** Summary of Goodness-of-Fit Statistics for Multigroup Invariance Tests for the Acquired Capability for Suicide Scale**–**Fearlessness about Death

Model no.	Description	Contrast	χ^2^	df	χ^2^ Δ	df Δ	Δ p	CMIN/df	CFI	Δ CFI	SRMR	RMSEA	pClose
1	Configural model (unconstrained model)		21.355	12				1.780	0.996		0.0212	0.034	0.860
2	Measurement model (factor loadings constrained equal	2 vs. 1	28.732	18	7.377	6	0.287	1.596	0.995	0.001	0.0289	0.030	0.956
3	Scalar invariance (factor loadings and intercepts constrained to be equal)	3 vs. 2	53.321	24	24.589	6	0.001	2.222	0.987	0.008	0.0283	0.043	0.767
**4**	**Residual invariance (factor loadings, intercepts, error co-variances, and error residuals constrained to be equal)**^a^	**4 vs. 3**	**84.573**	**33**	**31.252**	**9**	**<0.001**	**2.563**	**0.977**	**0.010**	**0.0324**	**0.048**	**0.568**

Bold indicates the non-invariant model (error co-variance and error residuals significantly different between groups).

^a^ Tests of error co-variances and error residuals for each individual negatively worded item of the ACSS**–**FAD showed non-significant degradation in model fit, suggesting strict invariance (same between groups).

## Discussion

This study compared the measurement properties of the INQ-10 and the ACSS**–**FAD in autistic and non-autistic adults to assess their appropriateness for measuring the ITS proximal risk factors for suicide in autistic adults. This is the first time that the scales of a well-validated suicide theory have been compared in autistic and non-autistic people. We reported configural and metric non-invariance in the thwarted belonging and burdensomeness subscales of the INQ, whereas a modified ACSS**–**FAD met criteria for scalar invariance, with negatively worded items meeting criteria for strict invariance. This will allow us to make informed comparisons of suicide mechanisms between autistic and non-autistic people.

Overall, results suggest that the INQ-10 operates differently in autistic adults compared with non-autistic adults. Configural non-invariance suggests that the latent constructs are experienced differently by autistic and non-autistic people. Viewed alongside our data screening information, the INQ may capture experiences—such as feeling socially isolated or experiencing low self-worth—that frequently occur for autistic people rather than the hypothesized rare experiences proposed by the ITS.^7^ Consistent with our hypotheses, there was evidence of metric non-invariance for the burdensomeness subscale, with each individual item indicating metric non-invariance, suggesting that autistic people interpret these items differently from non-autistic people. This could suggest that autistic people had difficulty interpreting and responding to items that required them to infer the mental states of others—such as attributing feelings of being “happier” or “better off” to the “people in my life”—in line with well-established literature describing differences in theory of mind among autistic people.^20^ Overall, this subscale cannot provide a comparable measure of burdensomeness between autistic and non-autistic adults. Future research could consider how autistic people experience burdensomeness and specifically whether other latent constructs, such as self-worth and agitation, may be relevant for autistic people. Clinicians should be aware that burdensomeness may be experienced and communicated differently by autistic people but that it does represent a risk factor.

Consistent with our hypothesis, there was evidence of metric non-invariance for the thwarted belonging subscale. In line with our hypothesis, the item “I often feel like an outsider in social gatherings” was metric non-invariant, indicating that autistic people interpret this item differently from non-autistic people. This supports the proposal of our steering group that feeling uncomfortable in social gatherings may be a core experience of autistic people, rather than an indicator of non-typical social isolation. Clinicians should take account of personal social preferences of autistic individuals when assessing risk. Surprisingly, items that contained abstract concepts, such as “disconnected” and “I belong,” demonstrated metric invariance, suggesting that these items were interpreted similarly by both autistic and non-autistic people. This could reflect reports that autistic people experience similar social needs to non-autistic people.^38^ Thus, these two items, along with the item describing satisfaction with the number and quality of friends could be compared between autistic and non-autistic people. Future research could explore *how* autistic people experience belonging and social connection in general and as protective factors.

We reported that, contrary to our hypotheses, there was evidence of scalar invariance in a modified ACSS**–**FAD, with evidence for strict invariance of negatively worded items. This suggests that non-concrete language (“not at all” and “very much”) and negative response options did not hinder autistic people any more than non-autistic people in choosing the correct response. Other researchers^39^ have reported similar response difficulties in non-autistic groups, which could suggest that the scale may benefit from revision. Any revision should consider the broader suggestion that the single construct of a reduced fear of death may be too narrow to reflect the changes that enable a suicide attempt: Clinical advice recommends broad screening for past painful and frightening experiences to identify possible suicidal capability,^40^ and recent innovations include a broader Acquired Capability with Rehearsal for Suicide scale.^41^ Future research could consider how these constructs are experienced by autistic people and the guidance required by clinicians for accurate risk assessment.

This study has several strengths. It is the first study to explore the measurement properties of self-report scales of a well-established suicide theory in a large sample of autistic adults and compare the responses with a matched sample of non-autistic adults. This is vital to inform how suicide assessment tools may need to be tailored to enable clinicians to accurately identify risk in autistic people. This study also has limitations, including reliance on self-report autism diagnosis. Variance between groups could be due to other confounds, such as higher prevalence of neurodevelopmental conditions in the autistic group, which could be explored in future research. This could also include exploring how autistic individuals with intellectual disability experience and express proximal risk factors for suicide.

In conclusion, this study reported that scores on the INQ-10 cannot be meaningfully compared between autistic and non-autistic people. However, with one item removed scores on the ACSS**–**FAD are comparable between these groups. Burdensomeness and thwarted belonging may represent proximal risk factors for suicide in autistic people but may be experienced and expressed differently in autistic compared with non-autistic people. Clinically, this suggests that tailored measurement tools and specific training may be required to identify risk and target interventions for autistic people.

## Notes

^*^ This study focusses on the experience of autistic adults without intellectual disability. This population has been reported to be at increased risk of suicide.^2^
